# Editorial: Deciphering the bone marrow microenvironment in hematologic malignancies

**DOI:** 10.3389/fonc.2023.1231467

**Published:** 2023-06-19

**Authors:** Manja Wobus, Martin Bornhäuser

**Affiliations:** Department of Medicine I, University Hospital Carl Gustav Carus, Technische Universität, Dresden, Germany

**Keywords:** acute myeloid leukemia, myelodysplastic syndromes, bone marrow, extracellular matrix, extracellular vesicles

Hematologic malignancies such as myelodysplastic syndromes (MDS), acute leukemias of the lymphoid (acute lymphoblastic leukemia, ALL) or myeloid (acute myeloid leukemia, AML) lineage arise from hematopoietic stem or progenitor cells (HSPCs) in the bone marrow (BM). Despite often achieving complete remission with induction chemotherapy, several patients will experience relapse, which remains a significant barrier to cure. Relapse-initiating cells are maintained by *in vivo* niches within the bone marrow (BM), thus, to evade and survive chemotherapy. The BM microenvironment/niche is composed of various cell types including mesenchymal stromal cells (MSCs), endothelial, perivascular, osteolineage, and neuronal cells (1–3). Alterations in the niche cause disruption of the homeostatic balance and foster hematologic diseases. Malignant cells can alter their microenvironment and create a leukemic niche advantaging abnormal cells at the expense of normal hematopoiesis. A better understanding of the complex underlying mechanisms may beneficial for new therapeutical strategies targeting the BM niche.

This Research Topic collected the latest findings and achievements in the field of basic and translational research into the malignant bone marrow microenvironment. It includes studies of extracellular cellular components such as vesicles and matrix, metabolic alterations of the malignant BM niche as well as of lymphoid aggregates.

This Research Topic consists of four manuscripts, including two original papers, one methods paper, and one mini review.

The review by Maynard et al. provides data about AML-derived changes in the BM niche, which enable enhanced metabolism of leukemic cells. While normal HSPCs mainly acquire ATP through glycolysis, malignant cells switch to the oxidative phosphorylation and TCA cycles to maintain their higher energy demands. These processes allow AML cells to maximise their ATP production, using multiple metabolites and fuelling rapid cell turnover which is a hallmark of the disease. The paper discusses future challenges with a view to understanding how AML cells are able to hijack metabolic pathways of niche cells and how new therapeutic targets can be elucidated.

Lang et al. compared different extracellular vesicles (EV) isolation methods from human AML BM cells and cell lines. They tested the efficacy and functional assay compatibility of four different EV isolation methods in leukemia-derived EVs: (1) membrane affinity-based: exoEasy Kit alone and (2) in combination with Amicon filtration; (3) precipitation: ExoQuick-TC; and (4) ultracentrifugation (UC). EVs were successfully isolated with all methods, as evidenced by highly maintained spherical- and cup-shaped vesicles in transmission electron microscopy. Nanoparticle tracking analysis of EV particle size and concentration revealed significant differences in EV isolation efficacy, with the exoEasy Kit providing the highest EV yield recovery. Of note, functional assays with exoEasy Kit-isolated EVs showed signficant toxicity towards treated target cells, e.g., MSCs, which was abrogated when combining exoEasy Kit with Amicon filtration. Additionally, MSCs treated with green fluorescent protein (GFP)-tagged exoEasy Kit-isolated EVs did not show any EV uptake, while EV isolation by precipitation demonstrated efficient EV internalization.

The cheapest method (UC) resulted in contaminated and destroyed EV fractions, while the isolation method with the highest EV yield (exoEasy Kit) appeared to be incompatible with functional assays. The authors identified two methods (precipitation-based ExoQuick-TC and membrane affinity-based exoEasy Kit combined with Amicon filtration) that yield pure and intact Evs and are also suitable for application in functional assays.

This study highlights the importance of selecting the right EV isolation method depending on the desired experimental design.

Another important component of the BM niche is the extracellular matrix (ECM), which was investigated by Bains et al. They performed a comparative analysis of *in vitro* deposited MSC-derived ECM from different MDS subtypes (lower-risk, LR, and higher-risk, HR) and healthy controls. Atomic force microscopy analyses demonstrated that MDS ECM was significantly thicker and more compliant than that from healthy MSCs. Scanning electron microscopy showed a dense meshwork of fibrillar bundles connected by numerous smaller structures that span the distance between fibers in MDS ECM. Glycosaminoglycan (GAG) structures were detectable in high abundance in MDS ECM as white, sponge-like arrays on top of the fibrillar network. The presence of higher concentrations of sulfated GAGs in MDS ECM was confirmed by different methods, such as fluorescent lectin staining with wheat germ agglutinin and peanut agglutinin and the Blyscan assay. Moreover, increased amounts of hyaluronic acid (HA) in the matrix of MSCs from LR-MDS patients were found to correlate with enhanced HA synthase 1 mRNA expression in these cells. CD34+ HSPCs displayed impaired differentiation potential after cultivation on MDS ECM and modified morphology, accompanied by decreased integrin expression, which mediates cell–matrix interaction.

In summary, Bains et al. provide evidence for structural alterations of the MSC-derived ECM in both LR- and HR-MDS. GAGs may play an important role in these remodeling processes during the malignant transformation, which leads to the observed disturbance in the support of normal hematopoiesis.

Book et al. investigated lymphoid aggregates (LA), which are occasionally seen in BM biopsies of MDS patients treated at the Tel Aviv Sourasky Medical Center, with the aim of evaluating their incidence and association with prognosis. LA polyclonality was detected by CD20/CD3 staining. MDS patients with LA were younger than those without LA. There was a trend towards poor prognostic parameters, including lower Hb, WBC, and platelets, higher LDH, BM cellularity, and IPSS-R score. The 1-year survival rate of LA+ patients was lower compared to LA− patients (70.6% vs. 83.0%). However, this trend could not be confirmed after 2 years.

The authors conclude that LA is relatively common (24%) in MDS BM biopsies, which might reflect the involvement of the immune system and poor prognosis. Future studies are planned to examine larger patient cohorts in order to clarify the incidence, significance and the pathophysiology of LA.

Taken together, the brief overview of the articles presented here in this Research Topic provides new insights into current research activities in the malignant BM niche, which could potentially open up further preclinical innovations.

## Author contributions

All authors listed have made a substantial, direct, and intellectual contribution to the work and approved it for publication.

## References

[1] Kfoury Y Scadden DT. Mesenchymal cell contributions to the stem cell niche. Cell Stem Cell (2015) 16:239–53. doi: 10.1016/j.stem.2015.02.019 25748931

[2] Pinho S Frenette PS. Haematopoietic stem cell activity and interactions with the niche. Nat Rev Mol Cell Biol (2019) 20:303–20. doi: 10.1038/s41580-019-0103-9 PMC6483843 30745579

[3] Kokkaliaris KD. Dissecting the spatial bone marrow microenvironment of hematopoietic stem cells. Curr Opin Oncol (2020) 32:154–61. doi: 10.1097/CCO.0000000000000605 32022758

